# Reconstruction of epidemic curves for pandemic influenza A (H1N1) 2009 at city and sub-city levels

**DOI:** 10.1186/1743-422X-7-321

**Published:** 2010-11-16

**Authors:** Shui Shan Lee, Ngai Sze Wong

**Affiliations:** 1 Stanley Ho Centre for Emerging Infectious Diseases, The Chinese University of Hong Kong, Shatin, Hong Kong

## Abstract

To better describe the epidemiology of influenza at local level, the time course of pandemic influenza A (H1N1) 2009 in the city of Hong Kong was reconstructed from notification data after decomposition procedure and time series analysis. GIS (geographic information system) methodology was incorporated for assessing spatial variation. Between May and September 2009, a total of 24415 cases were successfully geocoded, out of 25473 (95.8%) reports in the original dataset. The reconstructed epidemic curve was characterized by a small initial peak, a nadir followed by rapid rise to the ultimate plateau. The full course of the epidemic had lasted for about 6 months. Despite the small geographic area of only 1000 Km^2^, distinctive spatial variation was observed in the configuration of the curves across 6 geographic regions. With the relatively uniform physical and climatic environment within Hong Kong, the temporo-spatial variability of influenza spread could only be explained by the heterogeneous population structure and mobility patterns. Our study illustrated how an epidemic curve could be reconstructed using regularly collected surveillance data, which would be useful in informing intervention at local levels.

## Findings

The time course of an infectious disease epidemic is one important piece of information for understanding the dynamics of pathogen transmission. For a localized outbreak, for example, food poisoning, an epidemic curve is often conveniently drawn during case investigation. Describing the time course of a country-wide epidemic is more complex, which is not uncommonly complicated by reporting delay, discrepant access to diagnostics, varied public perception and the influence of accompanying health-seeking behaviours. In time of an emerging pandemic, these obstacles pose a great challenge to our society, when a timely construction of an epidemic curve is desirable. The spread of pandemic (H1N1) 2009 was a case in point. When the pandemic first hit the population, most people were non-immune to the novel virus, albeit the presence of partial immunity in some older people[1]. The relative lack of airborne transmission implies that the dissemination of the virus could be shaped largely by population structures, their networking pattern and human mobility[2]. An epidemic curve, if constructed, should reflect these characteristics for supporting the design of effective public health control programs.

Because of the spatial variability of the population, it is hypothesized that the epidemic curves could vary significantly from place to place. In this study we set out to describe the time course of the H1N1 epidemic with a spatial context in Hong Kong, a South-Eastern Chinese territory of about 1000 Km^2 ^in area. Since the diagnosis of the first case on 1 May 2009, all laboratory confirmed cases of pandemic (H1N1) 2009 were reported to the Government. Through the Centre for Health Protection, an anonymised dataset was obtained for the study, which included the age, gender and residential building location of each confirmed case. The residential address was transformed to x and y coordinates in Hong Kong Grid 1980 projection system. Geographically, Hong Kong can be divided into 18 districts and 400 District Council Constituency Areas (DCCA), each of the latter having an average of 17000 population for electoral purpose. ArcGIS version 9.2 was used for spatial exploration while time series analysis was performed to track the time course of the epidemic. A filtering procedure was applied to decompose the series into trend, seasonal and residual components (STL - seasonal trend decomposition procedure based on Loess), implemented on R[3]. Institutional approval for access to the data was obtained from HKSAR Department of Health, in compliance with the Personal Data (Privacy) Ordinance. Individual consent was deemed unnecessary in the analysis of collected surveillance data which did not involve primary data collection.

**Figure 1:**
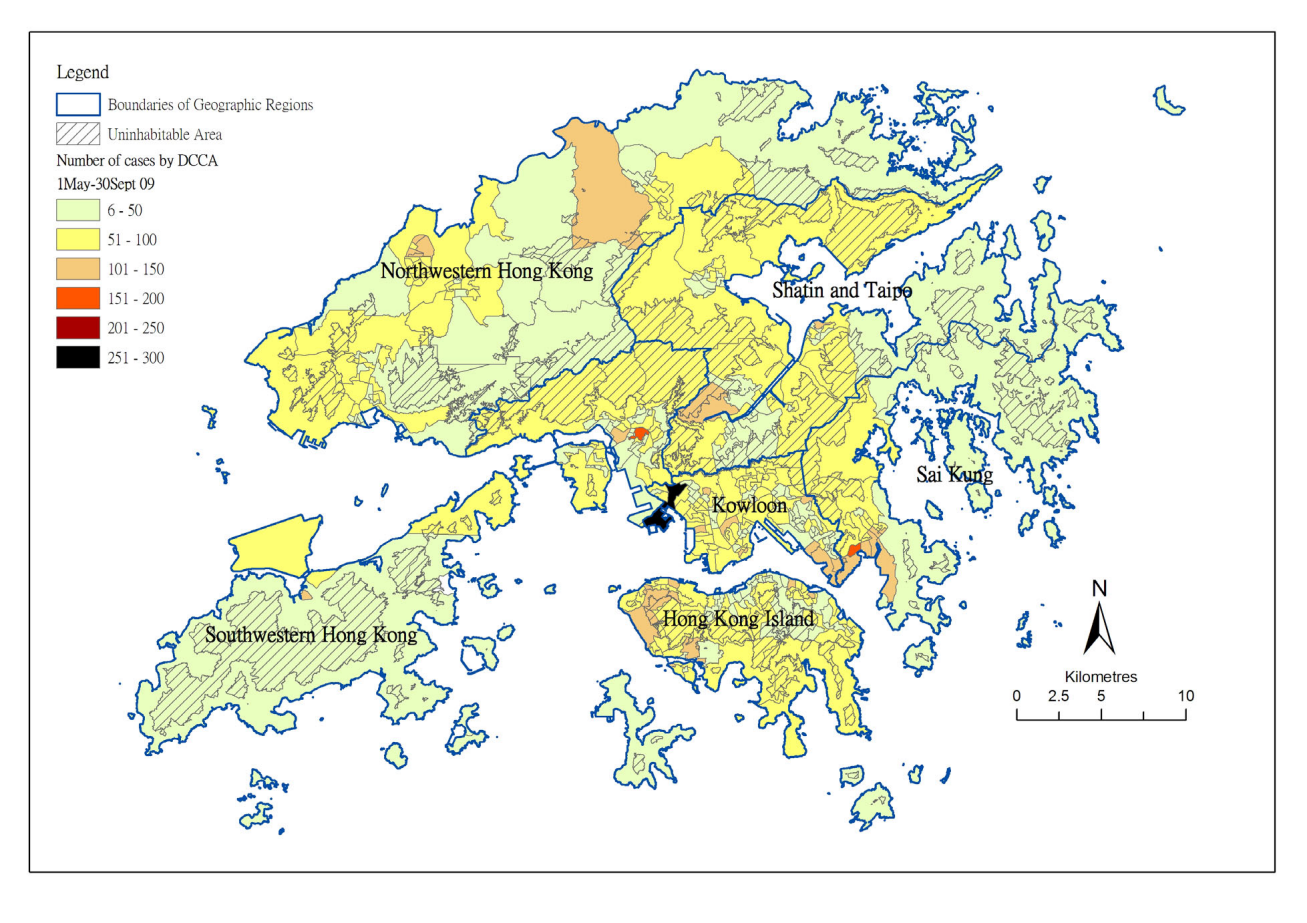
**Spatial distribution of reported Pandemic influenza (H1N1) 2009 cases in Hong Kong from May to September 2009, by district council constituency area (DCCA)**. Uninhabitable areas, including land elevated over 200 meters and water bodies, are hashed. Table 1 displays the population characteristics and statistics on reported cases for the 6 geographic regions - Hong Kong Island, Kowloon, North West, Sai Kung, Shatin/Tai Po, South West, the boundaries of which are given in thick lines.

Overall, a total of 24415 pandemic (H1N1) 2009 cases were successfully geocoded, out of 25473 (95.8%) reported between May and September 2009. The male-to-female ratio was 1.07:1. There was marked heterogeneity in the geographic spread of the reported cases, ranging from 6 cases to 272 cases per DCCA (figure 1). Evaluating at district level, the number of reported cases ranged from below 30 to > 50 per 100,000 populations. In the absence of physical boundaries between geographic units, people are free to move within and across districts and DCCA in their daily activity. We redefined six geographic regions representing places separated by natural borders like mountains and water bodies, after exclusion of uninhabitable areas. The region boundaries, population size and demographic characteristics of pandemic influenza (H1N1) 2009 cases are given in figure 1 and Table 1. The attack rate, expressed as the reported number per 100 resident population was similar across all regions, despite the difference in case density. The proportion of students (defined as people of age 5-19) in the reported case was higher than adults (age 20-64), a pattern opposite to that in the general population(Table 2).

**Table 1:** Population characteristics by geographic region (2006 by-census data) in Hong Kong

Geographic regions	**Area (km**^**2**^**)**	Population	**Population density per km**^**2**^	Proportional distribution			
				Infant (%) age 0-4	Student (%) age 5-19	Adult (%) age 20-64	Elderly (%) age > 64
Hong Kong Island	82.00	1302720	15886	2.96	14.96	68.47	13.69
Kowloon Peninsula	43.80	2019533	46106	2.86	15.63	65.66	15.85
Shatin & Taipo	133.57	901086	6746	2.79	17.54	69.65	10.02
Sai Kung	106.56	406442	3814	3.79	18.58	69.37	8.25
North West	297.49	1316957	4427	3.38	20.16	67.58	8.89
South West	140.94	914542	6489	3.42	17.18	67.49	12.44

**Table 2:** Characteristics of Pandemic influenza A (H1N1) cases by geographic region (May-September 2009) in Hong Kong

Geographic regions	Attack rate (%)	No. of cases	**Case density per km**^**2**^	Proportional distribution			
				Infant (%) aged 0-4	Student (%) aged 5-19	Adult (%) aged 20-64	Elder (%) aged > 64
Hong Kong Island	0.41	5288	64	9.21	56.88	32.98	0.93
Kowloon Peninsula	0.37	7412	169	10.85	54.13	34.04	0.98
Shatin & Taipo	0.32	2908	22	10.73	51.41	36.38	1.48
Sai Kung	0.38	1562	15	12.04	56.53	30.73	0.70
North West	0.34	4464	15	12.52	53.72	32.91	0.85
South West	0.30	2781	20	12.94	54.80	31.28	0.97

**Figure 2:**
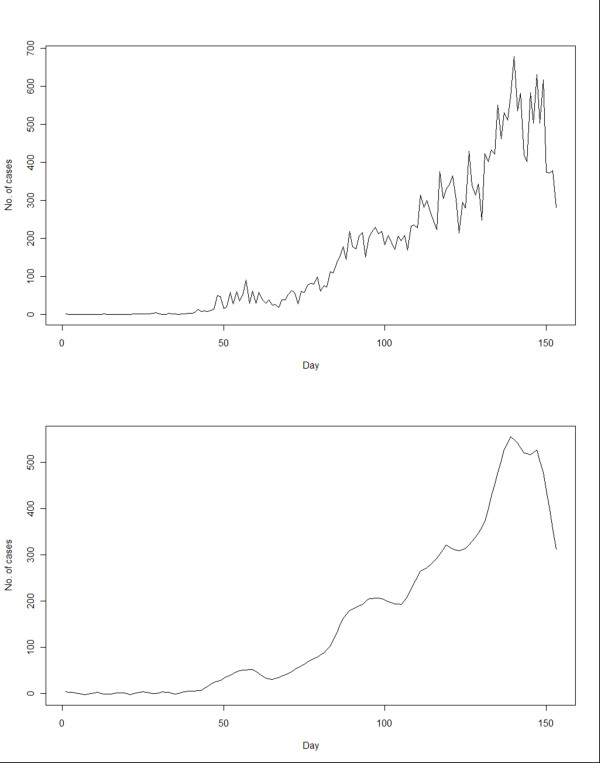
**Construction of epidemic curves using the original data (upper panel) and then by time series analysis after seasonal decomposition of time series by Loess (STL) and smoothing (lower panel)**. The procedure involved five steps of, firstly, detrending; secondly, cycle-subseries smoothing by loess; thirdly, low-pass filtering by moving average; fourthly, detrending by subtracting seasonal component; and finally, deseasonalizing.

**Figure 3:**
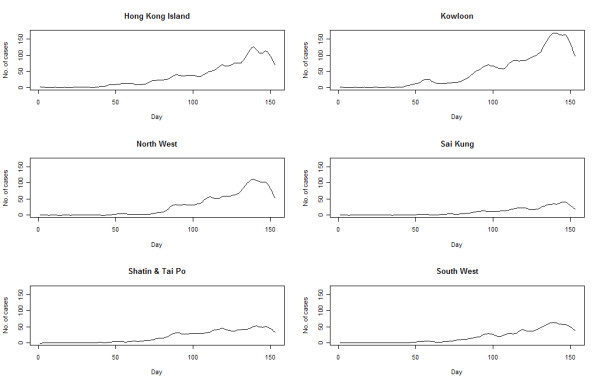
**Epidemic curves for Pandemic influenza (H1N1) 2009 by geographic regions - Hong Kong Island, Kowloon, North West, Sai Kung, Shatin/Tai Po, South West**.

**Figure 4:**
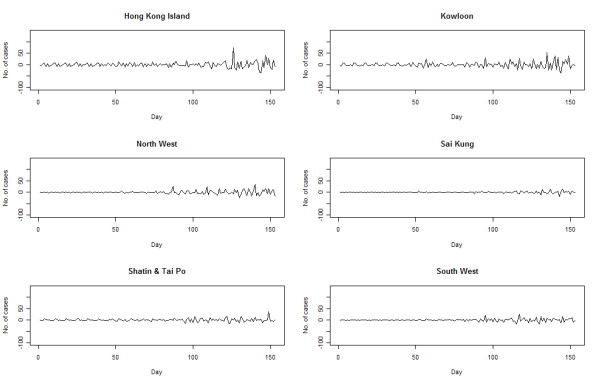
**Pattern of the residuals after seasonal decomposition of time series by Loess (STL) for the 6 geographic regions - Hong Kong Island, Kowloon, North West, Sai Kung, Shatin/Tai Po, South West**.

An epidemic curve was drawn from the reported numbers in the original dataset (Figure 2 - upper panel). In this study, the parameters and components of STL function (Y_t _= T_t_+S_t_+R_t _) were: t as the time unit, from 1 to 153; Y_t _as the daily count of H1N1 cases on day t; T_t _as the trend component; S_t _as the seasonal component, using 7-day as the smoothing window to account for the weekly cycles adopted by laboratories in the testing and reporting of results (tests on 3-day and 14-day windows were performed yielding less satisfactory results); and R_t _as the residual component. The final epidemic curve was reconstructed from the trend component after seasonal decomposition and the exclusion of residuals, through a sequence of operations employing Loess smoother. The Loess regression ĝ(x) smoothed y given x along the scale of the independent variable. The trend smoothing was computed in R by Loess[3]. The regression was locally weighted by $V_{i}(x)=W(\frac{\left|X_{i}-X\right|}{\lambda_{q}(x)})$ where W was the weighted function, λ_q_(x) was the *q*th farthest distance of x_i _from x, for i = 1 to n, with *q *as positive integer. The resultant trend (figure 2 lower panel) is characterized by a small peak at around Day 55-60, followed by a nadir and then rapid rise to the ultimate peak on Day 135. By defining students as those between the age of 5 and 19, the trend was plotted again using data on students alone and non-students, with the former bearing remarkable similarity to the all case series (results not shown). There were also marked variations across the 6 geographic regions in amplitude and configuration (figure 3). The early peak could only be seen in Kowloon, and less remarkably on Hong Kong Island region. The peak was reached in all 6 regions at around the same time, though the magnitude and the interval between onset and peak varied. The temporal profiles of residuals, constituted by the remains of the original dataset after seasonal and trend decomposition,[3] demonstrate that there were more spikes over time on Hong Kong Island and Kowloon Peninsula(figure 4).

Our study illustrated how an epidemic curve of an influenza epidemic could be reconstructed using regularly collected surveillance data, after decomposition of the time series. STL was applied on the assumption that the seasonal components had been contributed by weekly cycles of laboratory workloads, and that residuals reflected local outbreaks especially in schools. The smoothed trend therefore depicts the time course of the pandemic over a five-month period. The full time course of the epidemic could not be drawn as reporting of individual cases of pandemic (H1N1) 2009 ceased to be mandated at the end of September 2009. Interestingly, though, the peak appeared to have been reached before the reporting mechanism ended. A review of the epidemic curve constructed from influenza-like illness (ILI) surveillance suggested that this first wave came to the trough as of the end of October[4]. It is therefore quite likely that the full course of the epidemic has lasted for about 6 months.

Despite the small area of the territory of Hong Kong, spatial variation was observed in its initial diffusion[5]. This phenomenon was further characterized in this study by the demonstration of variability in: (a) the time point at which influenza cases were first introduced, (b) the time for a critical mass of cases to become cumulated, before the epidemic kicked off, and (c) the amplitude of the regional epidemics. Of note were the small peak and a subsequent nadir in the initial part of the time course, which have been attributed to mitigation introduced by the Government through school closure[6]. The initial small peak was however not seen in all geographic regions. This may be explained by the differential pattern of virus transmissions in each region in view of the variation of population structures, or that there were higher uptake of reported cases in some locations against the background of considerable public attention, when the news of an impending epidemic first broke out[7]. On the other hand, the landscape of the epidemic curve thus constructed was contributed largely by infections in students, an observation made in other studies on pandemic (H1N1) as well as seasonal influenza[8,9]. Against the background of a relatively uniform physical and climatic environment within Hong Kong, the temporo-spatial variability of influenza spread could only be explained by the heterogeneous population structure and mobility patterns.

Our study carried some limitations. Firstly we assumed that case reporting had been consistently executed over time. In the first five months, all clinically suspicious cases presenting to government clinical services and designated clinics were tested for the virus, alongside referrals from the private sector. While reporting can never be complete, the large number of cases reported (over 20,000) and the single public health agency supervising influenza surveillance in Hong Kong should have offset any inconsistency, thereby enhancing the robustness of the analysis. Secondly, STL decomposition was a filtering procedure based on an algorithm which may not have incorporated all determinants of the influenza transmission dynamics. The validity of the methods for field study would need to be further evaluated. Todate, STL has been used in syndromic surveillance, but with a different public health objective of detecting of outbreaks in the community[10]. In the development of an effective public health response, timeliness of the analysis and the use of regularly collected data are often crucial. In this connection, the algorithm described in this study has allowed the time course of a new epidemic to be drawn without resorting to sophisticated modeling techniques or simulations. The spatial variation in the time course of the pandemic (H1N1) 2009 was an important observation which may be further explored in context of strategies of public health interventions.

## Competing interests

The authors declare that they have no competing interests.

## Authors' contributions

SSL conceptualized the study, planned and coordinated the research, and wrote the first draft of the manuscript. NSW did the data exploration and conducted the analyses. Both approved the final version of the manuscript.
